# Safety, tolerability and pharmacokinetics of intravenous ghrelin for cancer-related anorexia/cachexia: a randomised, placebo-controlled, double-blind, double-crossover study

**DOI:** 10.1038/sj.bjc.6604148

**Published:** 2008-01-08

**Authors:** F Strasser, T A Lutz, M T Maeder, B Thuerlimann, D Bueche, M Tschöp, K Kaufmann, B Holst, M Brändle, R von Moos, R Demmer, T Cerny

**Affiliations:** 1 Oncological Palliative Medicine, Division Oncology/Haematology, Department Internal Medicine and Palliative Care Center, Department of IMD, Cantonal Hospital, Rorschacherstrasse, St Gallen 9007, Switzerland; 2 Institute of Veterinary Physiology, Vetsuisse Faculty University of Zurich and Zurich Center of Integrative Human Physiology, Winterthurerstrasse 260, Zurich 8057, Switzerland; 3 Division of Cardiology, University Hospital, Hebelstrasse 32, Basel 4031, Switzerland; 4 Division Oncology/Haematology, Department Internal Medicine, Cantonal Hospital, Rorschacherstrasse, St Gallen 9007, Switzerland; 5 Department Psychiatry, Obesity Research Centre, University of Cincinnati – Genome Research Institute, 2180 East Galbraith Road, Cincinnati, OH 45237, USA; 6 Laboratory for Molecular Pharmacology, The Panum Institute, University of Copenhagen, Blegdamsvej 3, Copenhagen DK-2200, Denmark; 7 Division of Endocrinology and Diabetes, Department Internal Medicine, Cantonal Hospital, Rorschacherstrasse, St Gallen 9007, Switzerland; 8 Division Oncology/Haematology, Department Internal Medicine, Cantonal Hospital, Loëstrasse 170, Chur 7000, Switzerland

**Keywords:** cachexia, anorexia, ghrelin, nutrition

## Abstract

Twenty-one adult patients were randomised to receive ghrelin on days 1 and 8 and placebo on days 4 and 11 or vice versa, given intravenously over a 60-min period before lunch: 10 received 2 *μ*g kg^−1^ (lower-dose) ghrelin; 11 received 8 *μ*g kg^−1^ (upper-dose) ghrelin. Active and total ghrelin, growth hormone (GH), and insulin-like growth factor 1 levels were monitored at baseline (4–5 days before day 1), during treatment days, and at end of study (day 17/18). Drug-related adverse events (assessed by NCI-CTC-toxicity criteria and cardiac examination) did not differ between ghrelin and placebo. No grade 3/4 toxicity or stimulation of tumour growth was observed. The peak increase of GH, a biological marker of ghrelin action, was 25 ng ml^−1^ with lower-dose and 42 ng ml^−1^ with upper-dose ghrelin. Morning fasting total ghrelin levels were higher (*P*<0.05) for upper-dose patients at end of study (3580 pg ml^−1^) than at baseline (990 pg ml^−1^). Insulin-like growth factor 1 levels did not change. At day 8, 81% of patients preferred ghrelin to placebo as against 63% at the end of study. Nutritional intake and eating-related symptoms, measured to explore preliminary efficacy, did not differ between ghrelin and placebo. Ghrelin is well tolerated and safe in patients with advanced cancer. For safety, tolerance, and patients' preference for treatment, no difference was observed between the lower- and upper-dose group.

Cancer patients often suffer from cancer anorexia/cachexia syndromes (CACS) and the consequences – fatigue, weakness, decreased performance status, poor tolerance of antineoplastic interventions, and psychosocial distress. Cancer anorexia/cachexia syndromes are characterised by a catabolic state triggered by tumour by-products, proinflammatory cytokines, and mediators of the neurohormonal system, causing loss of muscle and fat mass, anorexia, gastrointestinal dysmotility and early satiety, decreased anabolic drive, and hypermetabolism (Inui, 2002).

There are few pharmacological treatment options for CACS, but it has been shown that this patient population with advanced cancer has the capacity to respond to appetite stimulation therapy (Yavuzsen *et al*, 2005).

Ghrelin, an endogenous ligand for the growth hormone (GH) secretagogue receptor, displays dose-dependent GH-releasing activity (Kojima *et al*, 1999). Ghrelin, which is predominantly secreted by gastric endocrine cells, stimulates food intake and triggers a positive energy balance through a central mechanism involving hypothalamic neuropeptides. In preclinical cachexia models, ghrelin has had stimulatory effects on appetite and food intake (Hanada *et al*, 2003; Wang *et al*, 2006), lean body mass (DeBoer *et al*, 2007), gastrointestinal motility (Date *et al*, 2002), energy metabolism, and proinflammatory cytokine expression (Dixit *et al*, 2004), and it has also alleviated cancer chemotherapy-associated dyspepsia (Liu *et al*, 2006) and vomiting (Rudd *et al*, 2006). These experimentally induced cancer models provide promising but not sufficient evidence for an effect of ghrelin in human cancer, prompting clinical studies in a representative clinical population, including long-term studies in humans.

In human volunteers, intravenous (i.v.) (Nagaya *et al*, 2001a; Wren *et al*, 2001; Akamizu *et al*, 2004; Schmid *et al*, 2005; Levin *et al*, 2006) or subcutaneous (Enomoto *et al*, 2003; Druce *et al*, 2006) ghrelin showed safety and tolerability at dosages up to 10 *μ*g kg^−1^ – sufficient to promote orexigenic, prokinetic, and GH-releasing effects; in those studies, a sensation of warmth, sleepiness, bowel movements, and hunger were reported. Comparable results with i.v. ghrelin (single-dose bolus, daily for 3 weeks, or i.v. infusion) were reported in patients with chronic heart failure (Nagaya *et al*, 2001b, 2004), COPD (Nagaya *et al*, 2005), or diabetic gastroparesis (Murray *et al*, 2005).

In melanoma-bearing nude mice, ghrelin plasma concentration increased with cachexia progression (Hanada *et al*, 2004). In cancer patients with cachexia (various cancers, *n*=21 (Garcia *et al*, 2005); breast and colorectal cancer, *n*=18 (Wolf *et al*, 2006); lung cancer, *n*=21 (Shimizu *et al*, 2003)), ghrelin morning fasting levels were 1.3- to 1.5-fold higher than in those without cachexia and healthy controls. In contrast, ghrelin levels were normal in subgroups of patients in one study (39%) (Wolf *et al*, 2006) and all patients in another study (gastric and colorectal cancer, *n*=58) (Huang *et al*, 2007). Lower ghrelin values in patients with colorectal cancer (*n*=29) than in healthy controls (*n*=50) were also reported (D'Onghia *et al*, 2007). Preliminary findings suggest that pharmacological doses of ghrelin alleviate cancer cachexia. Tumour-bearing mice showed improved food intake and body composition only at a high intraperitoneal dose (40 *μ*g day^−1^) of ghrelin (Wang *et al*, 2006). In one pilot study, seven cancer patients had 31% higher energy intake with i.v. ghrelin than with placebo (5 pmol kg^−1^ min^−1^ for 180 min equals 3 *μ*g kg^−1^), with no adverse effects (Neary *et al*, 2004).

This trial was conducted to assess safety, tolerability, and pharmacokinetics in a 2-week trial of ghrelin infusion given intravenously, at one of two dose levels, once weekly, 1 h before lunch, to patients with far-advanced, incurable cancer, and involuntary loss of weight and appetite.

## MATERIALS AND METHODS

This single-centre, randomised, double-blind, placebo-controlled, two-arm, double-crossover study adhered to Good Clinical Practice (GCP) and the Declaration of Helsinki and was approved by the local Ethical Review Board and Health Authorities.

### Participants

Physicians at the Kantonsspital in St Gallen, Switzerland recruited adult patients with advanced incurable cancer who had loss of appetite (⩾3 visual analogue scale (VAS; 0=best, 10=worst)) and a weight loss of ⩾2% within 2 or ⩾5% within 6 months before the study not related to recent surgery.

Eligible patients gave written informed consent to participate, were able to eat without assistance, did not receive enteral or parenteral nutrition, and had no significant causes of secondary anorexia (defined as no severe symptoms or complications of the gastrointestinal tract impeding oral food intake) (Omlin and Strasser, 2007), as ensured by pre-baseline palliative oncology assessments. Patients were expected not to require new systemic antineoplastic treatment for the total study period of 3 weeks; those with unchanged continuous or weekly treatment for at least 2 months were eligible. Concomitant medication was to remain unchanged for at least 1 week before baseline. One patient having octreotide treatment was removed from analysis.

### Intervention

**Figure 1:**
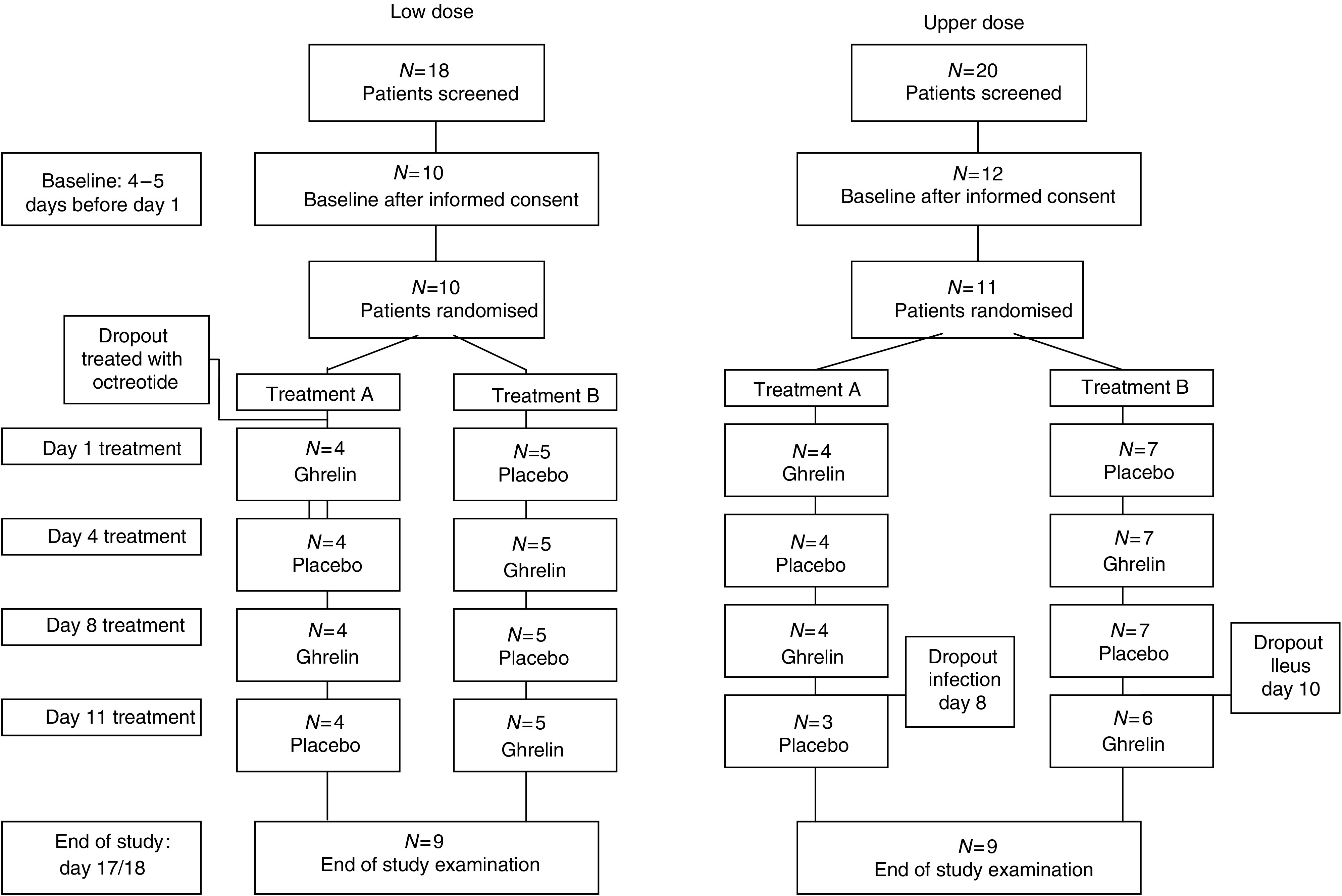
Flow of patients in a randomised, placebo-controlled, double-blind, double-crossover trial of i.v. ghrelin for cancer-related anorexia/cachexia.

In a randomised, double-blind, placebo-controlled, double-crossover trial, 4–5 days after baseline, patients received ghrelin on days 1 and 8 and placebo on days 4 and 11 or vice versa; end of study was day 17/18 (Figure 1).

On treatment days, patients ingested only water from midnight to breakfast. In the outpatient clinic, safety laboratory values and fasting hormone blood samples (including testosterone in male patients) were drawn at 0800 hours; venous access was maintained for pharmacokinetics. Patients received a standardised breakfast (120 g bread, 20 g butter, 60 g jam, 200 ml coffee with milk). At 1000 hours, a second i.v. line was inserted in the other arm for treatment given from 1030 to 1130 hours. Immediately after the end of the infusion, patients walked for approximately 5 min the 90 m to the restaurant where they received priority serving, starting the meal within 5 min. A hospital volunteer accompanied the patients and documented the conditions (i.e., quality of service, quiet atmosphere) at lunch. Following evaluations performed after lunch, all patients, except one inpatient, returned home.

Ghrelin of Good Manufacturing Practice quality was purchased from Clinalfa (Merck Biosciences, Switzerland) as vials of 88 *μ*g, stored at −20°C, and dissolved in 250 ml normal saline by the hospital pharmacy immediately before application. The treatment was titrated up within the first 10 min (20% increase each 2 min) and maintained for the next 50 min. The lower-dose group (LD) received 10 pmol kg^−1^ min^−1^ (equals 0.0336 *μ*g kg^−1^ min^−1^, approximately 2 *μ*g kg^−1^). The dose was based on the reported maximal GH stimulation in human volunteers (Wren *et al*, 2001) and multiplied by 2 to account for suspected ghrelin resistance (Shimizu *et al*, 2003). After observing treatment tolerance in the LD patients, we administered the upper-dose group (UD) 40 pmol kg^−1^ min^−1^ (approximately 8 *μ*g kg^−1^). Normal saline was used as placebo.

### Objectives

We tested the safety and tolerability of two dose levels of i.v. ghrelin in patients with far-advanced cancer based on toxicity, tumour measurements, and patients' perceived tolerance. We also assessed the pharmacokinetics.

### Outcome measures

Patients were assessed at each visit by using the NCI-CTC toxicity criteria Version 3.0, including standard blood examinations (hematology, chemistry panels), and cardiology evaluations, including echocardiography at baseline and at end of study. During the treatment phase, research personnel regularly contacted patients at home during the daytime to check for their safety. Before each infusion for each patient, the responsible investigator reviewed treatment logs for the preceding infusions and the results of the morning laboratory examinations. On study day 7 and at end of study, patients were asked about their perception of their tolerance and preference of day 1 (and 7) *vs* day 4 (and 11) treatments. Radiological measurements were made within 2 weeks before the first infusion and within 2 weeks after the last infusion by CT-scans, except for two patients with liver metastasis who had once an ultrasound (no. 11) or an MRI (no. 18), for one patient monitored only by ultrasound (no. 11) or once at study end (no. 4), one patient who had a prior MRI liver metastasis monitored by ultrasound, and one patient who had an ultrasound of the liver. An independent radiologist reviewed all films made before baseline, at baseline, and at end of study to judge tumour size and dynamics.

Patients' nutritional intake was monitored daily. At baseline, dieticians assessed patients' food preferences, reviewed the procedures and meals for the next 2 weeks, and distributed a food scale and standard protocols for prospective ‘third-person’ (family members of patients) assessments (Bruera *et al*, 1986). A trained volunteer accompanied patients at lunch in a designated quiet section of the hospital restaurant. Meals were photographed and kitchen personnel documented the weight of each meal component before and after each patient ate it.

To detect acute symptom effects of treatment, VAS assessments (0=best, 10=worst) of appetite, hunger, anxiety, early satiety, nausea, and fatigue were measured before, during, and after infusion. Immediately after lunch on treatment days, VAS assessments of the pleasantness of the meal, perceived appetite, and perception of amount of food intake were obtained.

For ghrelin assays, 5 ml blood was collected in a precooled EDTA vacutainer tube containing aprotinin (33 kIU), placed immediately on ice, and centrifuged (4°C, 3000 G, 5 min). For each millilitre of plasma, 10 *μ*l PMSF-Isopropanol, 50 *μ*l 1N-HCL, and 50 *μ*l aprotinin were added, and aliquots were stored at −80°C until batch analysis. Serum was collected and cooled for GH, insulin-like growth factor 1 (IGF-1), interleukin-6 (IL-6), and leptin analysis. Testosterone radioimmunoassays (Diagnostic Products Corporation; Bühlmann, Salzburg, Austria) were performed from serum sent to routine safety labs. The radioimmunoassay kits used for total and active ghrelin were from Linco Research (St Charles, MO, USA); for GH (active GH IRMA immunoradiometric assay) from Diagnostic Systems Laboratory (Webster, TX, USA); for IGF-1 (human, bovine) from Peninsula Laboratories (San Carlos, CA, USA); for IL-6 (Quantikine human ELISA) from R&D Systems (Minneapolis, MN, USA); and for leptin (human RIA) from Millipore (Billerica, MA, USA).

Autonomic dysfunction was assessed as described previously (Strasser *et al*, 2006). Standard deviation of beat-to-beat intervals (SDNN) was analysed at baseline and at end of study for 20 min in both the LD and UD; and in the UD, in addition, 30 min before infusion until after lunch on treatment days.

### Sample size

Safety and tolerability was assessed on the basis of a sample size of 10 patients per dose level.

### Treatment assignment, randomisation, and blinding

Patients were randomised by independent personnel at the hospital pharmacy, where the random allocation sequence produced (switches after 1 to maximal 3 patients) was assigned and the sealed envelopes for each patient distributed. A master randomisation list was kept in a locked container at the pharmacy. Copies of the documents in each sealed envelope were stored in a locked container accessible to clinicians for emergencies, as required by GCP standards.

Less than 30 min before each infusion, the pharmacy produced identical bags containing indistinguishable liquids of 250 ml normal saline with or without ghrelin.

The database was closed after completion of the study and rating of all adverse events. Thereafter, an independent senior physician who had controlled the randomisation procedure, the master randomisation list, and the broken envelopes revealed the treatment assignments.

### Statistical analyses

All analyses were performed with SPSS (Version 11.5). Descriptive statistics were used for demographic and baseline variables, frequencies of adverse events, and tumour measurements. For exploratory analyses of patients' preference of treatment, we used the exact binominal test. For pharmacokinetics (GH, ghrelin), glucose values, IGF-1 levels, nutritional intake, and SDNN, a comparison was made between changes from baseline for each individual subject between the two interventions (sum of two ghrelin periods *vs* sum of two placebo periods). For between-patient comparison of peak GH (median), the Wilcoxon's (no-signed) rank sum test (Mann–Whitney) was used; and for within-patient comparisons (ghrelin morning fasting levels 3 days after the prior ghrelin infusion, glucose, IGF-1, nutritional intake, and SDNN) the Wilcoxon's signed rank test was used.

## RESULTS

Flow (Figure 1) and demographics (Table 1) for the 20 patients studied were recorded. Oral intake at the fixed breakfast at treatment days was 296 kcal (stable disease (SD) 80) in LD and 276 kcal (118) in UD; one patient in LD and five in UD eat less than 250 kcal. Most patients (17 of 20) had ongoing inflammation (C-reactive protein (CRP) >10 mg ml^−1^) (Fearon *et al*, 2006). Creatinine was 83 *μ*mol l^−1^ (mean, SD 31) in LD and 73 *μ*mol l^−1^ (17) in UD and one patient each in LD and UP had a value above normal (<105 *μ*mol l^−1^). No patient had malignant gastroparesis. Two patients stopped study treatment early in the second week because of malignant bowel obstruction and blood-culture positive infection, respectively. Treatment of one patient was unblinded during the study because of apoplectiform deafness.

Concomitant pre-existing medications included laxatives (76%), opioids (67%), propulsive drugs (67%), antacids (62%), vitamins (57%), and many others. Three patients (upper dose only) were on unchanged treatment for >1 week with megestrol acetate (160 mg twice daily) and three different patients on intramuscular testosterone, and one patient each received fish oil (500 mg twice daily) or dexamethasone (8 mg day^−1^). Six patients (29%) received anticancer agents before and during the study (three gemcitabine; one each irinotecan, vinblastine, or bevacizumab). One patient was started on dexamethasone (4 mg twice daily) for liver capsule pain on day 14.

Of 205 adverse events, 49 possibly and 9 probably were related to an agent studied – placebo as well as ghrelin (Table 2). They included abnormal liver tests or low potassium (three patients on ghrelin, four on placebo); increased serum amylase, creatinine, and D-dimer (seven on placebo); and increased CRP (two on ghrelin). Blinded clinicians rated the other adverse events as unrelated or probably unrelated to treatment. Those were cardiac arrhythmia during LD ghrelin infusion; constipation or infection with UD ghrelin; sinus tachycardia, pulmonary rales, increased stool frequency, or back pain with LD placebo; and blurred vision with UD placebo. Body temperature and oxygen saturation remained unchanged during and after the infusion of ghrelin and placebo in both dosage groups. Of 13 serious adverse events, one – transient apoplectiform deafness on day 13 – was judged as probably related to treatment on day 11 (placebo).

The mean scores for tolerability of the study medication immediately after infusion and after lunch did not differ between ghrelin and placebo or between LD and UD. More patients preferred ghrelin to placebo (Table 3) at day 7 and at end of study, with no evidence of patients' awareness of their treatment assignment.

During the study period, two patients experienced progressive disease (PD). Before enrolment, one had had SD and one, PD. Of 16 patients with SD during the study period, 10 had PD, five had SD, and one had partial response before enrolment. Of two patients who stopped study treatment early, one had SD and one had PD at enrolment. The mean time interval between tumour assessments pre-baseline and at baseline was 79 days in LD and 29 days in UD, and between assessments at baseline and after the study was 34 and 25 days, respectively.

**Figure 2:**
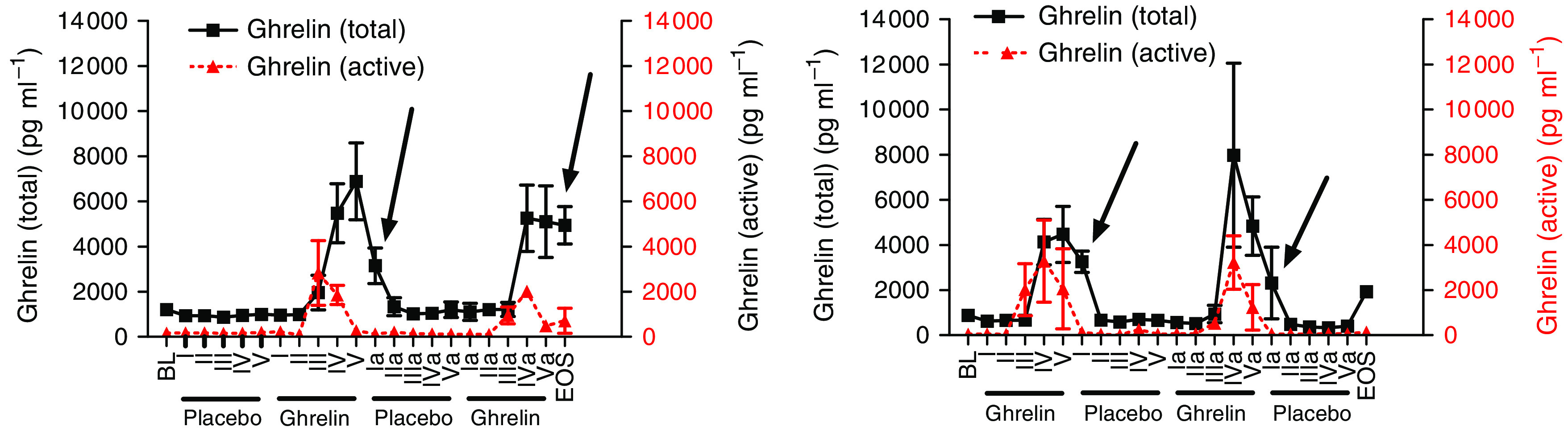
Pharmacokinetics of active and total ghrelin of the upper dose patients. BL, baseline; I-V, blood samples week 1 (I: morning fasting; II: immediately before ghrelin infusion (1030); III: during ghrelin infusion; IV: after ghrelin infusion (1130); V: after lunch (1230)); Ia-Va, blood samples week 2; EOS, end of study. *P*<0.001 for differences of morning fasting level of total ghrelin 3 days after ghrelin or placebo.

For total ghrelin, in the UD, elevated morning fasting levels were observed 3 days after the prior ghrelin infusion compared to after placebo (*P*<0.001), as confirmed by an independent, blinded laboratory (Figure 2).

The mean differences of the peak GH levels (of week 1 and week 2) compared to baseline were higher in UD (50 ng ml^−1^ (SD 20)) than in LD (28 ng ml^−1^ (6)) (*P*=0.004).

In one patient (UD) having gastrectomy 6 months before baseline, neither substantial differences in baseline values nor peak levels of active or total ghrelin or GH were detected.

Insulin-like growth factor 1 did not increase at day 17/18 as compared to study start in any patient examined (maximal increase from baseline was 170%); mean IGF-1 was 1359 pg per 100 *μ*l (±994) in LD (*n*=7) and 1096 pg per 100 *μ*l (±495) in UD (*n*=9), and mean change from baseline −2624 pg per 100 *μ*l (±2888) and −624 pg per 100 *μ*l (±962) (*P*=0.055).

During treatment days, blood glucose values compared to baseline after infusions were higher when patients received ghrelin than when receiving placebo in LD only after lunch (3.6 *vs* 2.5 mmol l^−1^ (*P*=0.005)) but not after infusion (1.5 *vs* 1.3 mmol l^−1^, *P*=0.16), in UD both after lunch (2.4 *vs* 1.3 mmol l^−1^ (*P*=0.01)) and after infusion (0.8 *vs* 0.2 mmol l^−1^ (*P*=0.044)).

Plasma levels of IL-6 did not change throughout the treatment period (results not shown).

There were no significant differences in nutritional intake or symptoms compared to baseline when patients received ghrelin or placebo. Nutritional-intake-at-lunch compared to baseline was in LD −105 kcal with ghrelin and −17 kcal with placebo, in UD 251 and 230 kcal, respectively; nutritional-intake-at-lunch-and-rest-of-the-day was (LD) 145 and 228 kcal, and (UD) 244 and 156 kcal, respectively (all *P*=NS). In UD patients not receiving concurrent chemotherapy (*n*=8), a trend towards increased differences compared to baseline for nutritional-intake-at-lunch-and-rest-of-the-day (ghrelin: 448 kcal; placebo: 128 kcal; *P*=0.093) but not nutritional-intake-at-lunch (ghrelin: 330 kcal; placebo: 200 kcal; *P*=ns) was observed.

Mean SDNN was 57±28 ms at baseline and 73±57 ms at end of study in 18 evaluable patients (*P*=ns); in UD (*n*=9), for ghrelin 84±40 ms and for placebo 78±35 ms in week 1 (*P*=NS), and in week 2, 75±35 and 80±27 ms (*P*=NS), respectively.

## DISCUSSION

This is, to our knowledge, the first trial investigating two doses of ghrelin in patients with advanced cancer and anorexia/cachexia. Intravenous ghrelin infusion for 60 min at 2 or 8 *μ*g kg^−1^ body weight is well tolerated and safe in these patients who represent a ‘real world’ population of cancer patients with anorexia/cachexia.

At present, no dose-limiting toxicity has been reported for ghrelin in animals or humans. The dosage used was based on the reported maximal GH stimulation in human volunteers (Wren *et al*, 2001) and on prior trials using up to 10 *μ*g kg^−1^ in healthy volunteers and 6 *μ*g kg^−1^ in patients (Nagaya *et al*, 2001b). Our data suggest a dose relationship with GH stimulation. In cachectic tumour-bearing mice (MCG101), higher ghrelin doses were required to increase food intake and body weight than in control mice (Wang *et al*, 2006). Other interventional CACS studies did not compare ghrelin doses (Hanada *et al*, 2003; Neary *et al*, 2004; DeBoer *et al*, 2007).

Morning fasting levels of ghrelin in patients (Shimizu *et al*, 2003; Garcia *et al*, 2005; Wolf *et al*, 2006; Huang *et al*, 2007) or animals (Hanada *et al*, 2004; Liu *et al*, 2006) with CACS are still poorly understood. In animals, both higher (Hanada *et al*, 2004) and lower (Liu *et al*, 2006) ghrelin levels than controls are reported. Several studies showed higher ghrelin levels (Shimizu *et al*, 2003; Garcia *et al*, 2005) or higher levels only in subgroups (61% of 18 breast and colorectal cancer patients) (Wolf *et al*, 2006) in patients with CACS as compared to non-cachetic cancer patients or healthy controls; however, normal (Huang *et al*, 2007) ghrelin levels were reported as well. The differences of fasting levels of ghrelin in cancer patients may be explained by differences in BMIs, 20.7 kg m^−2^ in LD and 20.6 kg m^−2^ in UD, and 18.5 kg m^−2^ in others (Shimizu *et al*, 2003). It remains unclear whether ghrelin plasma levels are increased in cancer patients and whether high plasma levels of ghrelin will induce resistance to ghrelin. It remains to be clearly shown whether the response to peripheral ghrelin differs depending on the prevailing ghrelin level.

In transgenic mice overexpressing ghrelin, the acute stimulation of food intake of exogenous ghrelin was not diminished (Wei *et al*, 2006). In patients with anorexia nervosa, in whom chronic hyperghrelinaemia presents with two- to three-fold increased levels (Broglio *et al*, 2004), i.v. ghrelin (1 *μ*g kg^−1^ per hour for 5 h (5 pmol kg^−1^ min^−1^ × 300 min; Miljic *et al*, 2006) or 1 *μ*g kg^−1^ (Broglio *et al*, 2004)) caused much lower GH and glucose increases than in healthy volunteers and caused no appetite stimulation but increased sleepiness. In two patients having ghrelin-producing tumours in gastro-entero-pancreatic system (Corbetta *et al*, 2003; Tsolakis *et al*, 2004), BMI remained high and the appetite good despite failure to respond to anticancer treatment.

In our study, we observed no major unexpected tumour-growth dynamics, but the study design with short intervals of the tumour measurements impedes firm conclusions. As ghrelin is also a potent GH secretagogue, there are concerns about GH-mediated stimulation of tumour growth, especially regarding treatment of cancer patients. Several cell lines express the ghrelin receptor (Yeh *et al*, 2005; Ekeblad *et al*, 2006) and secrete ghrelin (Yeh *et al*, 2005). *In vitro* studies suggest that ghrelin may enhance the proliferation of prostate (Yeh *et al*, 2005) and pancreatic (Duxbury *et al*, 2003) cancer cells, but not of a lung cancer cell line, where it induced dose-dependent inhibition of cell proliferation and increased apoptosis (Cassoni *et al*, 2006). Some tumours from archival samples express ghrelin (Jeffery *et al*, 2005), whereas others do not (Cassoni *et al*, 2006; Mottershead *et al*, 2007). Tumour incidence is not increased in patients with anorexia nervosa (Mellemkjaer *et al*, 2001) despite elevated ghrelin levels. The hepatic GH effector IGF-1 levels are not correspondingly increased in conditions with high endogenous plasma ghrelin levels, such as ghrelin-producing tumours (Corbetta *et al*, 2003; Tsolakis *et al*, 2004). Furthermore, the administration of ghrelin does not significantly affect the IGF-1 level in healthy volunteers (Enomoto *et al*, 2003), patients with cardiovascular (Nagaya *et al*, 2004) or pulmonary diseases (Nagaya *et al*, 2005), tumour-bearing animals (DeBoer *et al*, 2007), or our patients with CACS. In contrast, studies using synthetic oral ghrelin mimetics have shown a significant effect on the IGF-1 level in volunteers and in the frail elderly (Smith, 2005) or patients with cancer cachexia (Garcia *et al*, 2007).

Higher morning fasting total ghrelin levels 3 days after i.v. ghrelin administration suggest a carryover effect. As the half-life of ghrelin is short – approximately 15 min – a systematic mistake in the analysis was thought likely, but an independent, blinded laboratory confirmed our results. Ghrelin levels were normal before infusion at 1030 hours. Renal function was not impaired. These unexpected findings of total, but not active, ghrelin remain unexplained at present but may indeed be without any physiological significance.

Our study of patients with far-advanced cancer was not designed to detect effects on nutritional intake, eating-associated symptoms, or lean-body mass. We found no major differences for these efficacy parameters between ghrelin and placebo in preliminary analyses. Our finding contrasts with the data observed in animal models (Hanada *et al*, 2003; Wang *et al*, 2006; DeBoer *et al*, 2007). Our methodology with treatment of secondary anorexia, nutritionist-monitored lunch meals, full placebo control of all outcomes, and standardised procedures and time schedules makes systemic errors unlikely.

In contrast to one recent small series of patients with mainly (5/7) breast cancer (Neary *et al*, 2004), our patients had tumours typically leading to CACS. A high intrapatient variability of symptoms and nutritional intake is reported in patients with advanced, incurable cancer (Stromgren *et al*, 2006). Baseline food intake (Gilg and Lutz, 2006) and dietary patterns with high protein or liquid intake (Blom *et al*, 2006) may influence ghrelin regulation. Drugs for symptom control (eg, haloperidol (Jaszberenyi *et al*, 2006), 5-HT-3 antagonist (Depoortere *et al*, 2006)) may interact with ghrelin metabolism. Patients often have enhanced levels of proinflammatory cytokines and stress, which are reported to increase preprandial activation of ghrelin secretion (Kristenssson *et al*, 2006) by activation of sympathetic nerves but not by epinephrine (Mundinger *et al*, 2006). Alterations in testosterone levels may influence ghrelin regulation, as testosterone treatment in prepubertal boys decreased ghrelin values (Lebenthal *et al*, 2006). Patients may have remaining side effects of prior chemotherapy mediating CACS (Hutton *et al*, 2007).

Ghrelin may prolong the premature gastric phase III of migrating motor complex tone in the proximal stomach (Tack *et al*, 2006), leading to enhanced gastrointestinal motility without (Tack *et al*, 2006) and with (Liu *et al*, 2006) increased food intake, but some studies show no stimulatory effect of ghrelin on motility (Depoortere *et al*, 2006; Ohno *et al*, 2006). Patients with advanced cancer often have autonomic dysfunction (Strasser *et al*, 2006), as did 83% of our patients. We found no differences in autonomic function during or after two single infusions of ghrelin.

Some of our patients seem to prefer ghrelin to placebo, and this may be associated with the effect of peripheral ghrelin targeting the mesolimbic reward circuitry (Abizaid *et al*, 2006). The limitations of this study design include the lack of chronic efficacy data beyond two weekly infusions; namely, body composition (lean body mass, fat mass (Theander-Carrillo *et al*, 2006)), objective subconscious locomotive motor and physical activity (Jaszberenyi *et al*, 2006), energy expenditure measurements (Lejeune *et al*, 2006), and gastrointestinal motility (Binn *et al*, 2006; Blom *et al*, 2006). Our results on dose responsiveness may be influenced by unbalanced groups: UD patients had more metastases, greater weight loss, lower dietary intake, more early satiety, and were closer to death. However, given the double-crossover design, no effect on outcomes is expected from unequal (4 *vs* 7) randomisation in the UD group.

The patients in our study represent a very diverse population, since this trial was conducted with more or less unselected patients, with CACS having mostly far advanced cancer, as the median survival documents, and various tumour types. Our main aim was to explore the safety and tolerability of i.v. ghrelin in such a clinical situation, in which the interpretation of (negative) efficacy data requires considerable caution. In the two patients with stomach and oesophageal cancer, the clinical efficacy of ghrelin may be limited, since in patients having had vagotomy ghrelin induced only an increase in GH secretion but not in energy intake (le Roux *et al*, 2005). Given the foreseen clinical application, namely a relatively short interval between intervention and meal but not at the same time (difficult for patients to have continuous infusions during meals or to inject subcutaneously ‘real time’ during meals), we chose to offer lunch immediately after, but not during, the ghrelin infusion, and this time difference may explain the lack of difference in energy intake observed between ghrelin and saline. The safety and tolerability data support further exploration of the therapeutic potential of natural ghrelin, namely escalation of dose (Wang *et al*, 2006) and frequency and chronic administration. The patient population may be stratified for baseline ghrelin levels (Garcia *et al*, 2005; Wolf *et al*, 2006), and other factors need to be controlled for, namely genetic alterations of the ghrelin gene (Holst and Schwartz, 2006), cytokine levels (Dixit *et al*, 2004), stress level (Kristenssson *et al*, 2006), hypogonadism (Strasser *et al*, 2006), patients' eating preferences (Blom *et al*, 2006), baseline food intake (Gilg and Lutz, 2006), and gastric emptying (Binn *et al*, 2006). These strategies may counteract the series of many negative cachexia phase III trials (EPA, cannabinoids) or single not confirmed studies (ATP, thalidomide), treating uniformly all patients having loss of weight and appetite, despite promising pathophysiological concepts.

In conclusion, ghrelin administered intravenously as one therapeutic dose and repeated once after 1 week was safe and well tolerated by both LD and UD patients with far-advanced cancer and anorexia/cachexia. Several patients preferred ghrelin to placebo despite a lack of major differences in food intake or symptoms. The stimulation of GH, reflecting biological activity, was dose-dependent. Anorexia/cachexia remains a burdensome clinical problem with few treatment options. Further research with ghrelin will explore dose escalations, route and schedule modifications, and mechanisms of ghrelin resistance.

## Figures and Tables

**Table 1:** Demographics of 21 patients with cancer-related anorexia/cachexia

	**Lower-dose group (*n*=9)**	**Upper-dose group (*n*=11)**
*Age (years)*		
Median (min, max)	66 (45, 73)	70 (45, 80)
		
*Gender*		
Female/male	1/8	2/9
		
*Diagnosis*		
Pancreatic cancer	1	3
Mesothelioma	2	0
Prostate cancer	1	2
Colorectal cancer	3	1
Stomach/esophageal cancer	0	2
NSCLC	1	2
Urogenital cancer	1	0
Cholangiocarcinoma	0	1
		
*Metastasis (all patients ⩾1; none CNS)*		
Liver	4	6
Lung	4	2
Bone	3	6
Peritoneal	2	4
Lymph node	1	8
		
*Survival time (days)*		
Median (min, max)	233 (14, 436)	67 (16, 386)
		
*Prior chemotherapy (number of regimens)*		
0	3	3
1	1	4
2	3	2
3–5	2	2
		
Prior radiation therapy	3	5
Prior hormonal therapy	1	2
		
*Weight (kg)*		
Median (min, max)	59 (54, 95)	54 (44, 77)
		
*Body mass index (kg m*^−*2*^)		
Median (min, max)	21.7 (15.7, 30)	20.6 (17.3, 30.4)
		
*Weight loss last 2 months* (%)		
Median (min, max)	3.6 (2.2, 15.1)	6.0 (3.6, 11.5)^a^
		
*Nutritional intake at lunch (kcal)*		
Median (min, max)	650 (144, 1133)	304 (179, 700)
		
*Nutritional intake, whole day (kcal)*		
Median (min, max)	1237 (222, 1864)	889 (179, 1876)
		
*Appetite (0*=*best, 10*=*worst)*		
Median (min, max)	60 (7, 80)	71 (14, 89)
		
*Early satiety (0*=*no, 10*=*worst)*		
Median (min, max)	25 (2, 79)	52 (10, 95)
		
*Heart rate before 1st infusion (BPM)*		
Median (min, max)	90 (50, 103)	83 (54, 120)
		
*C-reactive protein (mg ml*^−*1*^)		
Median (min, max)	25 (2, 178)	24 (3, 145)
		
*Free testosterone (pmol l*^−*1*^)^b^		
Median (min, max)	22.4 (2.3, 170)	12.6 (<0.5, 25)
		
*Ghrelin, total (pg ml*^−*1*^)		
Median (min, max)	1041 (317, 1416)	1015 (533, 2598)
		
*Ghrelin, active (pg ml*^−*1*^)		
Median (min, max)	121 (24, 322)	102 (11, 250)
		
*Leptin (ng ml*^−*1*^)		
Median (min, max)	0.8 (0.4, 6.4)	1.9 (0.3, 7.4)
		
*Growth hormone (pg ml*^−*1*^)		
Median (min, max)	1.2 (0.3, 4.9)	1 (0.3, 3.8)
		
*IGF-1 (pg per 100 μl)* c		
Median (min, max)	3968 (808, 9143)	1820 (1121, 3675)
		
*Plasma glucose (mmol l*^−*1*^)		
Median (min, max)	4.8 (4.1, 7.5)	5.6 (4.4, 7.9)
		
*Prior major gastrointestinal surgery*		
Gastrectomy	0	1
Whipple procedure	0	1

NSCLC=non-small cell lung cancer; CNS=central nervous system; BPM=beats per minute; IGF-1=insulin-like growth factor 1.

a Two patients had missing data on weight loss 2 months before study entry, but had weight loss 6 months before study entry of 8 and 11.6%, respectively.

b Testosterone levels are reported only for men (LD *n*=8, UD *n*=9).

c IGF-1 levels are reported for LD *n*=8 and UD *n*=11.

**Table 2:** Adverse events of treatment with intravenous ghrelin in 21 patients with cancer-related anorexia/cachexia

	**Lower-dose group (*n*=9)**		**Upper-dose group (*n*=11)**	
**Adverse events related to study drug (probable or possible, all NCI-CTC grade 1 or 2)**	**Ghrelin**	**Placebo**	**Ghrelin**	**Placebo**
*During infusion on treatment days*				
Increased bowel activity^a^	3	5	5	3
Abdominal pain			3	
Dry mouth			3	1
Worsening of pre-existing neuropathy			1	
Dizziness				1
Shortness of breath (overeaten, aspiration)^b^	1	1		
Chest pain				
Nausea		1		
Increased stool frequency		1		
Sweating	2			
Asthenia			1	
				
*Between infusion days*				
Abdominal pain			2	
Apoplectiform deafness		1		
Diarrhea			1	1
Nausea			1	
Vomiting^b^	1	2		
Constipation		1		

NCI-CTC=common toxicity criteria (CTC) established by the National Cancer Institute (NCI).

a In five patients both on ghrelin and placebo.

b In one patient both on ghrelin and placebo.

**Table 3:** Patients' preference of treatment between placebo and intravenous ghrelin

	**Day 7**				**End-of-study**			
	**Lower dose (*n*=9)**		**Upper dose (*n*=11)**		**Lower dose (*n*=9)**		**Upper dose (*n*=9)**	
	**G-P^a^**	**P-G^a^**	**G-P^a^**	**P-G^a^**	**G-P-G-P^a^**	**P-G-P-G^a^**	**G-P-G-P^a^**	**P-G-P-G^a^**
Treatment preference (VAS), median (min, max)^b^	42 (3, 66)	80 (49, 100)	45 (2, 96)	77 (54, 100)	20 (10, 99)	54 (5, 100)	69 (56, 94)	83 (56, 100)
Preference for ghrelin (cutoff VAS 50),^c^ number (%)	7 (78%)^d^		9 (82%)^d^		6 (67%)^d^		6 (60%)^d^	

a Treatment sequence (G=Ghrelin, P=Placebo).

b Visual Analogue Scale (VAS: 0–100), low numbers indicate that the patient prefers first (day 7) and third (end-of-study) treatment, high numbers second and fourth treatment, respectively.

c VAS treatment preference score <50 in patients receiving the Ghrelin-Placebo-Ghrelin-Placebo order, 100-VAS score >50 in patients receiving the Placebo-Ghrelin-Placebo-Ghrelin order.

d Exact binominal two-sided tests: 4: *P*=NS, 5: *P*=0.065, 6: *P*=NS.
